# *Ndufa6* regulates adipogenic differentiation via *Scd1*

**DOI:** 10.1080/21623945.2021.2007590

**Published:** 2021-12-07

**Authors:** Jingwei Zhang, Wei Wang, Ninghan Feng, Xuan Jiang, Shenglong Zhu, Yong Q Chen

**Affiliations:** a Department of metabolism, Wuxi School of Medicine, Jiangnan University, Wuxi, China; b Department of Urology the Wuxi No. 2 People's Hospital, Wuxi 214002 China; c Wuxi Translational Medicine Research Center and Jiangsu Translational Medicine Research Institute, Wuxi, China

**Keywords:** Obesity, adipogenic differentiation, *Ndufa6*, *Scd*

## Abstract

Obesity and associated complications are becoming a pandemic. Inhibiting adipogenesis is an important intervention for the treatment of obesity. Despite intensive investigations, numerous mechanistic aspects of adipogenesis remain unclear, and many potential therapeutic targets have yet to be discovered. Transcriptomics and lipidomics approaches were used to explore the functional genes regulating adipogenic differentiation and the potential mechanism in OP9 cells and adipose-derived stem cells. In this study, we found that NADH:ubiquinone oxidoreductase subunit A6 (*Ndufa6*) participates in the regulation of adipogenic differentiation. Furthermore, we show that the effect of Ndufa6 is mediated through stearoyl-CoA desaturase 1 (*Scd1*) and demonstrate the inhibitory effect of a SCD1 inhibitor on adipogenesis. Our study broadens the understanding of adipogenic differentiation and offers NDUFA6-SCD1 as a potential therapeutic target for the treatment of obesity.

## Introduction

Epidemiological investigations have shown that the prevalence of obesity has increased dramatically worldwide. Increased obesity contributes to four million deaths annually and 120 million disability‐adjusted life‐years [1]. Therefore, the prevention, reduction and treatment of obesity and its complications are urgent issues for public health [2,3].

Obesity is characterized by imbalanced food intake and energy expenditure [4]. Excessive energy is stored in the form of triglycerides, leading to an augmented adipocyte number and/or size [5]. In addition, aberrant adipogenic differentiation plays a critical role in the progression of obesity [6]. Adipogenic differentiation is the process that converts preadipocytes into adipocytes, and this process is accompanied by fat synthesis and follow-up lipid droplet formation and enlargement [7]. Differentiation of preadipocytes into adipocytes occurs through various mechanisms. In mammalian cells, there are three main adipogenesis regulators: peroxisome proliferator-activated receptor γ (PPARγ), CCAAT/enhancer binding protein α (C/EBPα) and sterol regulatory element-binding protein (SREBP-1) [8]. C/EBPs are expressed in the early differentiation phase and activate PPARγ expression [9]. PPARγ promotes lipogenic gene expression, and SREBP-1 controls fatty acid (FA) biosynthesis, such as acetyl-CoA carboxylase and fatty acid synthase [10]. Fatty acid binding protein 4 (FABP4), adiponectin, and fatty acid synthase (FASN) are responsible for the formation of mature adipocytes. FABP4 is mainly expressed in adipocyte tissue and is known to promote the storage of lipids [11]. Adiponectin is an adipokine that is secreted by adipocytes, and it is involved in the crosstalk between adipose tissue and other metabolic tissues [12]. FASN is an enzyme involved in endogenous fatty acid synthesis and is considered a central enzyme in this process [13]. In contrast, a transcription factor, preadipocyte factor-1 (PREF-1), is downregulated during adipogenic differentiation [14]. Therefore, adipogenic differentiation depends on the coordinated regulation of gene expression.

Inhibiting key adipogenic genes may be an effective antiobesity therapeutic strategy. Indeed, several inhibitors targeting fat synthesis enzymes (e.g., FASN and acetyl-CoA carboxylases or ACC) have been shown to exert therapeutic effects in obesity and related metabolic disorders [15–19]. However, the adipogenic differentiation process involves numerous pathways, and many potential targets for suppressing fat synthesis have yet to be discovered.

In the present study, we conducted transcriptomics analyses in two classical adipogenic differentiation models. We found that NADH:ubiquinone oxidoreductase subunit A6 (Ndufa6) participates in the regulation of adipogenic differentiation. Furthermore, we show that the effect of Ndufa6 is mediated through stearoyl-CoA desaturase 1 (Scd1). Thus, the newly discovered NDUFA6-SCD1 pathway may serve as an attractive therapeutic target for obesity.

## Results

### Ndufa6 *is upregulated in adipogenic differentiationx*

**Figure 1.**
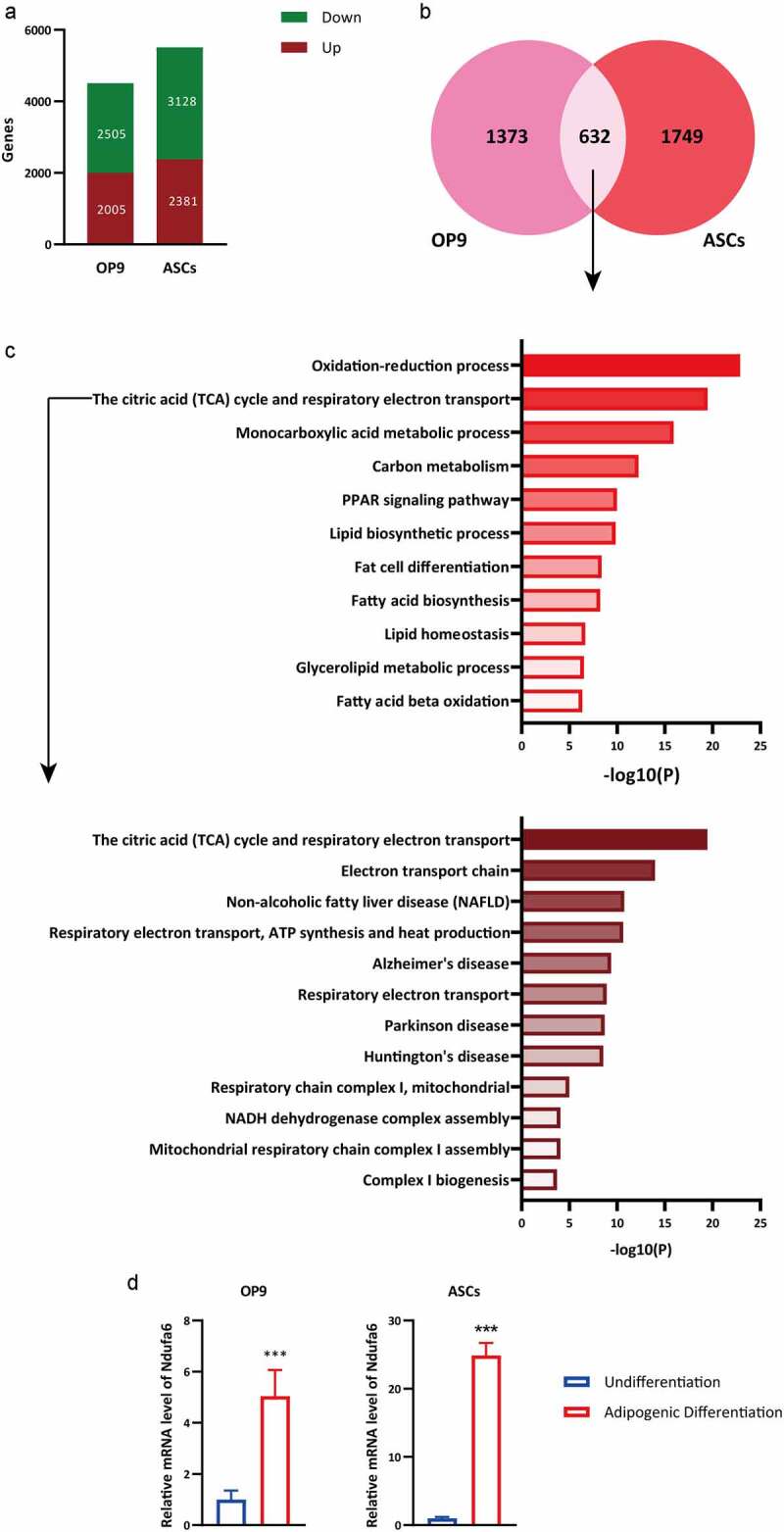
**RNA-Seq analysis of undifferentiated and differentiated OP9 and ASCs**. RNA-Seq data were obtained from OP9 cells and ASCs with or without adipogenic differentiation. (a) The numbers of up- and downregulated genes in OP9 and ASCs after adipogenic differentiation were selected. (b) The Venn diagram illustrates the number of upregulated genes and common upregulated genes in OP9 and ACSs after adipogenic differentiation. (c) GO analysis of common upregulated genes in OP9 and ASCs after adipogenic differentiation. GO, gene ontology. (d) The relative mRNA level of *Ndufa6* in OP9 cells and ASCs with or without adipogenic differentiation was tested with RT–qPCR (n = 3). Values are presented as the mean ± standard deviation. Statistical analysis was performed by the LSD t-test. * P < 0.05; ** P < 0.01; *** P < 0.005

To uncover potential genes that are involved in the regulation of adipogenic differentiation, transcriptomics was performed comparing differentiated to undifferentiated OP9 or differentiated to undifferentiated ASC. There were 2005 and 2381 genes upregulated after adipogenic differentiation in OP9 and ASC, respectively (Figure 1a). Among the two upregulated gene sets, 632 genes were the same (Figure 1b). Subsequently, these genes were subjected to Gene Ontology (GO) analysis, and the top two terms were ‘oxidation–reduction process’ and ‘TCA cycle and respiratory electron transport’ (Figure 1c). This result indicated that adipogenic differentiation was accomplished by mitochondrial oxidative metabolism. Electron transport is an important step in mitochondrial oxidative metabolism. Thus, the GO term ‘TCA cycle and respiratory electron transport’ was subdivided, and the results presented a wealth of terms related to complex I (Figure 1c). Complex I is the largest mitochondrial respiratory chain complex, and its deficiency accounts for almost one-third of respiratory chain disorders [20]. To validate this analysis and identify novel candidate genes related to adipogenic differentiation, the top 10% of the 632 upregulated genes were selected. Most genes were already known to participate in adipogenic differentiation, such as *Adipoq* (4800-fold in OP9 and 780-fold in ASC) and *Fabp4* (3400-fold in OP9 and 74-fold in ASC). Among the top-induced genes that had not been previously studied in adipogenic differentiation, we focused on *Ndufa6*, a subunit of complex I that is essential for correct complex assembly [21]. Its expression was upregulated >20-fold in ASCs and >5-fold in OP9 cells after adipogenic differentiation, as measured by qPCR (Figure 1d).

### Ndufa6 *silencing prevents adipogenic differentiation*

**Figure 2.**
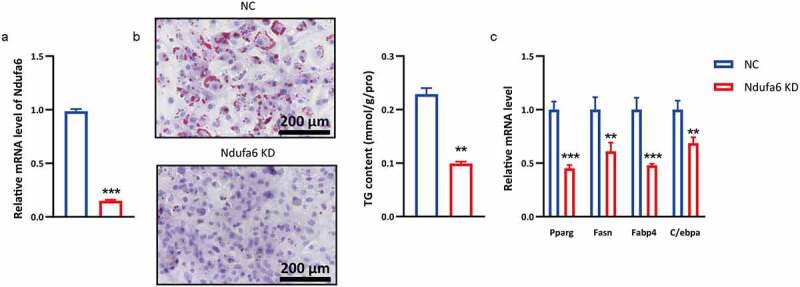
***Ndufa6* knockdown inhibits adipogenesis**. Before adipogenic differentiation, OP9 cells were transfected with *Ndufa6* siRNA (Ndufa6 KD) or control siRNA (NC), and 24 h later, cells were incubated with 1 mM rosiglitazone to induce adipogenic differentiation. (a) RT–qPCR was used to test the *Ndufa6* silencing efficiency in OP9 cells. (b) Oil red O staining was performed after adipogenic differentiation. The lipid droplets were stained red, and nuclei were stained blue by haematoxylin. The scale bar in the figure is 200 μm. The cellular TG content was tested in OP9 cells after adipogenic differentiation (n = 3), and the test results were normalized by the protein content. (c) The relative mRNA levels of adipogenic differentiation marker genes were tested with RT–qPCR (n = 3). Values are presented as the mean ± standard deviation. Statistical analysis was performed by the LSD t-test. * P < 0.05; ** P < 0.01; *** P < 0.005

OP9 cells have the advantages of rapid adipogenic differentiation and high transfection efficiency [22]. To uncover its role in adipogenic differentiation, *Ndufa6* was silenced during adipogenic differentiation. A siRNA targeting *Ndufa6* substantially downregulated *Ndufa6* gene expression in OP9 cells (Figure 2a). Cells transfected with *Ndufa6* siRNA had markedly smaller lipid droplets and lower TG content than cells transfected with control siRNA (Figure 2b). Consistently, 4 adipogenic differentiation marker genes, *Pparg, Fasn, Fabp4* and *C/ebpα*, were significantly downregulated in *Ndufa6*-silenced cells (Figure 2c). These data indicate that *Ndufa6* silencing prevents adipogenic differentiation in OP9 cells.

### Ndufa6 *inhibition decreases the level of monounsaturated fatty acids*

**Figure 3.**
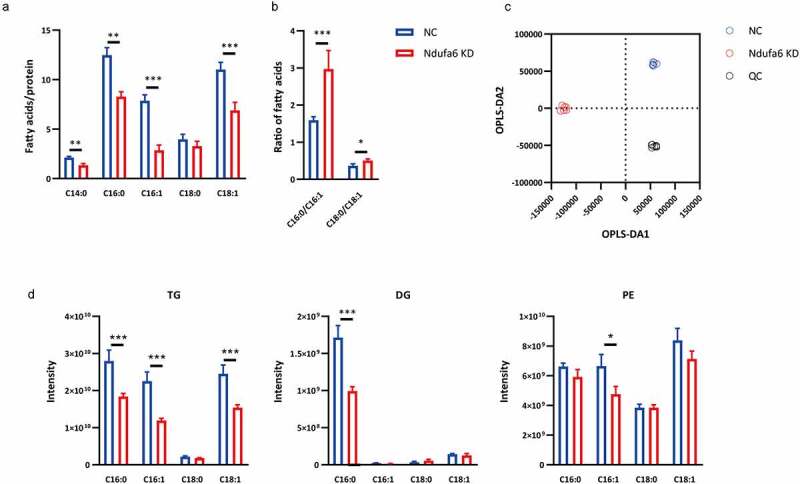
**Lipidomic analysis**. After adipogenic differentiation, OP9 cells with (Ndufa6 KD) or without (NC) *Ndufa6* knockdown were collected, and cellular fatty acids and lipidomics were performed. (a) Determination of cellular fatty acids after adipogenic differentiation by GC–MS and (b) the ratio of *Scd1* product to substrate (C16:0/C16:1, C18:0/C18:1) (n = 6). (c) OPLS-DA score plot of lipids derived from OP9 cells after adipogenic differentiation. Lipidomics was performed by LC–MS in the negative ion mode and positive ion mode (n = 6). (d) Under the positive ion mode, the composition of selected fatty acids (C16:0, C16:1, C18:0 and C18:1) in lipids was determined. Statistical analysis was performed by the LSD t-test. * P < 0.05; ** P < 0.01; *** P < 0.005

To determine which types of lipids were decreased in *Ndufa6* knockdown cells, FAME and lipidomics were performed. The levels of C14:0, C16:0, C16:1 and C18:1 were decreased in *Ndufa6* knockdown cells compared to control cells (Figure 3a), as determined by FAME. Most noticeably, the ratios of C16:0 to C16:1 and C18:0 to C18:1 were significantly increased (Figure 3b), indicating a reduced synthesis of monounsaturated fatty acids (MUFAs), possibly due to reduced stearoyl-CoA desaturase (SCD) activity. An orthogonal partial least square discrimination analysis (OPLS-DA) plot showed significant differences between the control (NC) and *Ndufa6* knockdown groups [Q2(cum) = 0.798] (Figure 3c). C16:1 and C18:1 fatty acid levels were mainly decreased in triacylglycerol (TG) and phosphatidylethanolamine (PE), while the C16:0 level was reduced in TG and diacylglycerol (DG) (Figure 3d). No alterations in other fatty acids or lipids were found (Fig. S1). There are 4 SCD isoforms (Scd1-Scd4) in mice and 2 isoforms (SCD1 and SCD5) in humans [23]. Scd1 is a rate-limiting enzyme that converts saturated fatty acids (SFAs) into MUFAs, mainly C16:1 and C18:1 [24]. The results suggested that Scd1 may mediate the adipogenesis suppression caused by *Ndufa6* knockdown.

### *Transcriptomics analysis implicates* Scd1 *as a target of* Ndufa6

**Figure 4.**
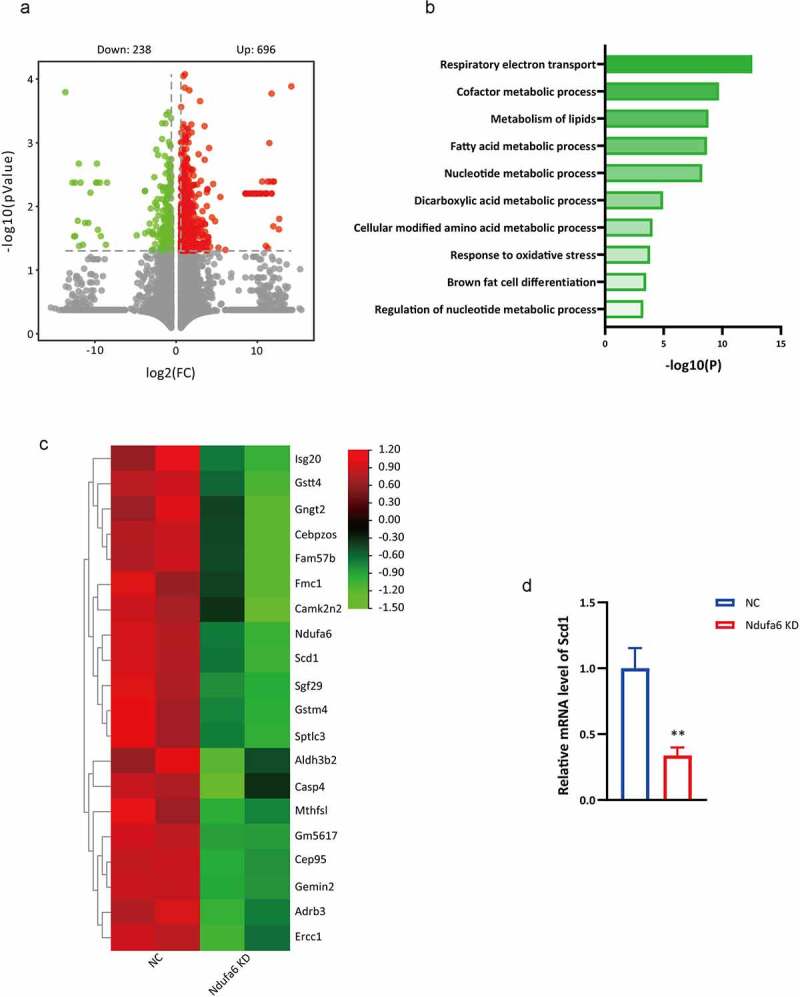
**Transcriptomics data**. After adipogenic differentiation, OP9 cells with or without *Ndufa6* knockdown were collected, and transcriptomics was performed. (a) Volcano plot of the differentially expressed genes (DEGs) in OP9 with (Ndufa6 KD) or without (NC) *Ndufa6* knockdown after adipogenic differentiation. (b) GO analysis of downregulated genes. (c) The heatmap displays the top 20 downregulated genes in *Ndufa6* knockdown cells. (d) The relative mRNA level of *Scd1* in OP9 cells after adipogenic differentiation (n = 3). Statistical analysis was performed by the LSD t-test. * P < 0.05; ** P < 0.01; *** P < 0.005

To explore the potential mechanism of *Ndufa6* in adipogenic differentiation, gene expression profiles were determined by RNA-seq in OP9 cells with or without *Ndufa6* knockdown. A total of 934 differentially expressed genes (≥1.5-fold change, p value<0.05) were identified, of which 238 were downregulated and 696 were upregulated (Figure 4a). Significantly enriched functional categories, such as respiratory electron transport and lipid and fatty acid metabolic processes, were observed among the downregulated genes (Figure 4b). However, no specifically enriched biological processes were found among the upregulated genes. A heatmap of the top 20 downregulated genes was plotted (Figure 4c), and *Ndufa6* and *Scd1* had similar expression patterns. Decreased *Scd1* expression in *Ndufa6* knockdown cells was confirmed by RT–qPCR (Figure 4d). The expression of *Scd2, Scd3* and *Scd4* was not detectable in OP9 cells. Transcriptomics data corroborate the lipidomics results, implicating *Scd1* as a target of *Ndufa6*.

### Ndufa6 *knockdown inhibits adipogenic differentiation through* Scd1

**Figure 5.**
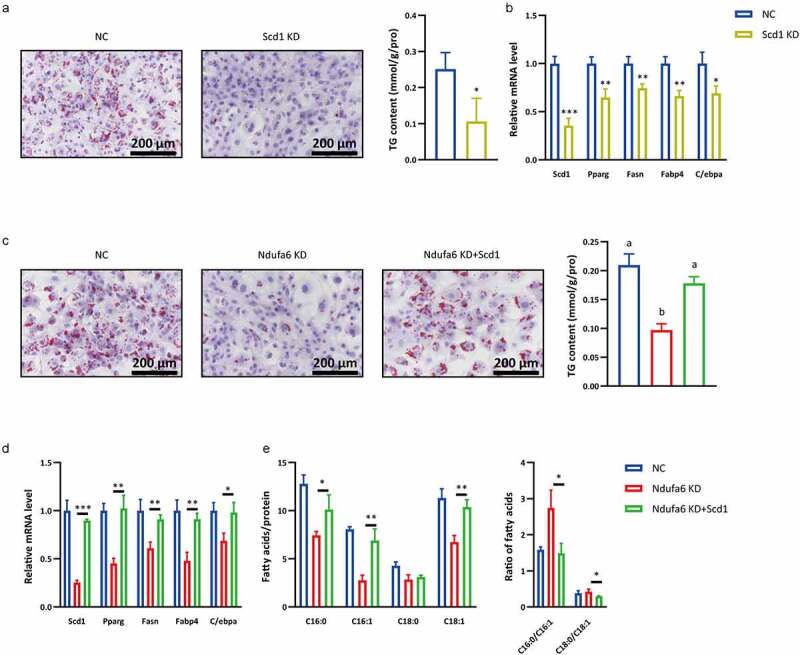
***Scd1* participates in *Ndufa6*-mediated adipogenic differentiation**. Before adipogenic differentiation, OP9 cells were transfected with *Scd1* siRNA (Scd1 KD) or control siRNA (NC), and 24 h later, cells were incubated with 1 mM rosiglitazone to induce adipogenic differentiation. (a) Oil red O staining was performed after adipogenic differentiation. The lipid droplets were stained red, and nuclei were stained blue by haematoxylin. The scale bar in the figure is 200 μm. The cellular TG content was tested in OP9 cells after adipogenic differentiation (n = 3), and the test results were normalized by the protein content. (b) RT–qPCR was used to test the relative mRNA level of *Scd1* and other adipogenic differentiation marker genes in OP9 cells with or without *Scd1* knockdown after adipogenic differentiation (n = 3). (c) Before adipogenic differentiation, OP9 cells were transfected with *Ndufa6* siRNA (Ndufa6 KD) or *Ndufa6* siRNA and pcDNA3.1-*Scd1* vector (Ndufa6 KD+Scd1) or control siRNA and pcDNA3.1 (NC). Oil red O staining was performed after adipogenic differentiation. The lipid droplets were stained red, and nuclei were stained blue by haematoxylin. The scale bar in the figure is 200 μm. The cellular TG content was tested in OP9 cells after adipogenic differentiation (n = 3), and the test results were normalized by the protein content. (d) RT–qPCR was used to test the relative mRNA level of *Scd1* and other adipogenic differentiation marker genes (n = 3). (e) After adipogenic differentiation, the cellular fatty acids were tested, and the *Scd1* product and substrate (C16:0, C16:1, C18:0 and C18:1) were analysed (n = 3). Statistical analysis was performed by the LSD t-test and one-way ANOVA. * P < 0.05; ** P < 0.01; *** P < 0.005. Different letters represent significant differences (P < 0.05, ANOVA)

To investigate whether *Scd1* participates in *Ndufa6*-mediated adipogenic differentiation, *Scd1* was silenced or overexpressed in OP9 cells. When *Scd1* was silenced individually, markedly decreased lipid droplets, TG content (Figure 5a) and the mRNA levels of adipogenic genes (*Pparg, Fasn, Fabp4* and *C/ebpα*) were observed (Figure 5b). However, when *Scd1* was overexpressed and *Ndufa6* was knocked down, significantly increased lipid droplets, TG content (Figure 5c), adipogenic gene mRNA (Figure 5d) and C16:1 and C18:1 levels (Figure 5e) were observed. These results indicate that *Ndufa6* knockdown inhibits adipogenic differentiation through *Scd1*.

### Scd1 *inhibitor suppresses adipogenic differentiation*

**Figure 6.**
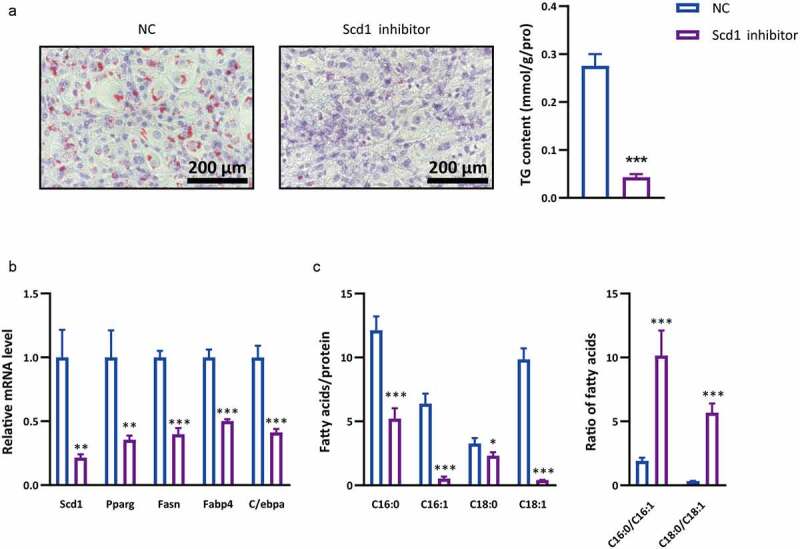
**Effects of *Scd1* inhibitor on adipogenic differentiation**. During adipogenic differentiation, OP9 cells were incubated with *Scd1* inhibitor or solvent control (NC). (a) Oil red O staining was performed after adipogenic differentiation. The lipid droplets were stained red, and nuclei were stained blue by haematoxylin. The scale bar in the figure is 200 μm. The cellular TG content was tested in OP9 cells after adipogenic differentiation (n = 3), and the test results were normalized by the protein content. (b) RT–qPCR was used to test the relative mRNA level of *Scd1* and other adipogenic differentiation marker genes in OP9 cells with or without *Scd1* inhibitor after adipogenic differentiation (n = 3). (c) After adipogenic differentiation, the cellular fatty acids were tested, and the *Scd1* product and substrate (C16:0, C16:1, C18:0 and C18:1) were analysed (n = 3). Statistical analysis was performed by the LSD t-test. * P < 0.05; ** P < 0.01; *** P < 0.005

The above results demonstrate that the *Ndufa6-Scd1* pathway may be a potential therapeutic target in obesity intervention. *Ndufa6* is a subunit of complex I, and its inhibition may induce unintended side effects, as a previous study demonstrated that complex I inhibition results in glycolysis enhancement and lactic acidosis [25]. Thus, we investigated whether a *Scd1* inhibitor alone could suppress adipogenic differentiation. The *Scd1* inhibitor A939572 markedly decreased lipid droplets, the cellular TG content (Figure 6a), the expression of adipogenic differentiation marker genes *Pparg, Fasn, Fabp4* and *C/ebpα* (Figure 6b) and the levels of C16:0, C16:1, C18:0 and C18:1 (Figure 6c).

## Discussion

In the present study, we found that *Ndufa6* is a key regulator in adipogenic differentiation. *Ndufa6* is a subunit of complex I, the largest and most complex enzyme in the oxidative phosphorylation system [21,26]. Oxidative phosphorylation is essential for normal adipogenic differentiation [27,28]. Complex I assembly errors or conformational changes impair the cellular oxidative phosphorylation system [20]. Notably, *Ndufa6* knockdown markedly inhibited adipogenic differentiation, whereas *Ndufa6* overexpression did not affect adipogenic differentiation (data not shown). Hence, *Ndufa6* likely exerts its role in adipogenic differentiation through assembled complex I but not itself. During adipogenic differentiation, the cells retained a high level of metabolic activity and increased substrate consumption along with a marked increase in the mitochondrial content and higher β-oxidation [29]. Lipid metabolism, including lipid transport, synthesis, and catabolism, requires high levels of energy and fully functional mitochondria, and the normal function of complex I is critical for these processes [30]. Therefore, it is anticipated that loss of other subunits of complex I will also inhibit adipogenic differentiation.

Our results suggested that Ndufa6 may be a therapeutic target for obesity. However, it should be noted that complex I dysfunction induced by inhibiting Ndufa6 may lead to impairment of the ability of the respiratory chain to oxidize NADH to NAD^+^ and the overproduction of ROS [21,31]. Excessive levels of ROS provoke lipid peroxidation and damage cellular proteins and DNA [32]. Therefore, on the basis of our study, the development and proper selection of Ndufa6 inhibitors may contribute to the treatment of obesity.

We identified *Scd1* as a target of *Ndufa6* in regulating adipogenic differentiation. *Ndufa6* knockdown prominently decreased MUFA levels in cells. This phenotype is consistent with the phenotype of *Scd1* knockdown in previous reports [33–35]. SCD1, a stearoyl-CoA desaturase converting stearic acid (C18:0) and palmitic acid (C16:0) into oleic acid (C18:1n9) and palmitoleic acid (16:1n7), respectively, requires electrons for the desaturation reaction [36]. Thus, it is likely that complex I dysfunction induced by *Ndufa6* knockdown leads to inhibition of SCD1 activity in addition to the downregulation of transcription. Numerous studies have revealed that blocking SCD1 leads to significant inhibition of adipogenesis [33,37–39]. Some metabolic diseases, such as obesity and non-alcoholic fatty liver disease, involve adipogenesis [24]. Thus, targeting SCD1 can be an effective treatment for these metabolic diseases. The results suggested that targeting *Ndufa6*, the upstream gene of SCD1, can also cause inhibitory effects of SCD1. This provides a new idea and a new way to develop SCD1 inhibitors in the future.

Other fatty acid desaturases also require electrons for the desaturation reaction. It is unclear why polyunsaturated fatty acid levels were not affected in *Ndufa6* knockdown cells (Fig. S1A).

Numerous studies have reported that key adipogenic genes, such as *Pparg, Fasn, Fabp4* and *C/ebpα*, directly participate in adipogenic differentiation [8,40,41]. *Pparg* and *C/ebpα* together promote differentiation by activating adipose-specific gene expression, such as *Fasn* and *Fabp4*, and by maintaining each other’s expression at high levels [40]. Knockdown of both *Ndufa6* and *Scd1* markedly decreased the expression of adipogenic marker genes, indicating that the *Ndufa6*–*Scd1* pathway may be an upstream regulator of adipogenic differentiation. Therefore, the *Ndufa6*–*Scd1* pathway can be a key therapeutic target for the management of obesity.

## Conclusions

In summary, we have uncovered a new function of *Ndufa6*, identified *Scd1* as a *Ndufa6* target gene in adipogenic differentiation, and demonstrated the inhibitory effect of a SCD1 inhibitor on adipogenesis. Our study broadens the understanding of adipogenic differentiation and provides potential therapeutic targets for the treatment of obesity.

## Materials and Methods

### Reagents

Minimum essential medium α (αMEM, ThermoFisher Scientific, 12561056), Dulbecco’s modified Eagle medium (DMEM, ThermoFisher Scientific, 11965092), Dulbecco’s modified Eagle medium/nutrient mixture F-12 (DMEM/F12, ThermoFisher Scientific, 11330032), foetal bovine serum (FBS, VivaCell Biotechnology, C04001), rosiglitazone (MedChemExpress, HY-17386), insulin (Shanghai Yuanye Bio-Technology, S12033), 3-isobutyl-1-methylxanthine (IBMX, Aladdin Biochemical Technology, I106812), epidermal growth factor (EGF, GenScript, Z02972), fibroblast growth factor-basic (bFGF, GenScript Biotech, Z03016), type I collagenase (Sangon Biotech, A004194), penicillin G sodium salt (Sangon Biotech, A600135), streptomycin sulphate (Sangon Biotech, A610494), Glutamax (ThermoFisher Scientific, 35050061), trypsin (Sangon Biotech, A100458), jetPRIME® transfection reagent (Polyplus transfection, 114–15), MolPure endo-free plasmid mini kit (Shanghai YEASEN Biotech, 19021ES), oil red O (Sangon Biotech, A600395), Alizarin Red S staining kit (Solarbio Life Science, G1452), A939572 (*Scd1* inhibitor, Sigma, SML2356), RIPA buffer (Beyotime, Biotechnology, P0013B), protease inhibitor cocktail (MCE, HY-K0010), Skim (BD, 232100), anti-NDUFA6 (Biodragon-immunotech, BD-PN0922), anti-SCD1 (ABclonal, A16429), anti-β-ACTIN (ABclonal, AC026), HRP goat anti-rabbit IgG (ABclonal, AS014), PVDF membranes (Millipore, ISEQ00010), ECL (Millipore, WBKLS0500), triglyceride (TG) assay kit (Nanjing Jiancheng Bioengineering Institute, A110-1-1), total protein assay kit (Nanjing Jiancheng Bioengineering Institute, A045-4-2), ultrapure RNA kit (CWBIO, CW0581M), HiScript® III 1st strand cDNA synthesis kit (Vazyme Biotech, R312-02), Hieff UNICON® qPCR SYBR green master mix (Shanghai YEASEN Biotech, 11198ES), second strand cDNA synthesis kit (Beyotime Biotech, D7172), and HIFI PCR mix for NGS (CWBIO, CW2648) were all commercially available. Tn5 transposase was purified according to a published protocol [42].

### Cell cultures and adipogenic differentiation

OP9 cells were maintained in αMEM supplemented with 5% FBS. For OP9 adipogenic differentiation, cells were grown to 90% confluence and then changed to adipogenic differentiation medium (DMEM supplemented with 5% FBS, 1 mM rosiglitazone) every three days for a total of 8 days. Adipose-derived stem cells (ASCs) were isolated according to a previous report [43] with slight modification. In brief, 8- to 10-week-old male C57/BL6J mice were sacrificed, and subcutaneous adipose tissues were taken from the inguinal fat pads. Next, tissues were washed extensively with phosphate-buffered saline (PBS) containing 2% penicillin/streptomycin. Upon removal of debris, the tissues were minced with scissors and digested with 0.1% type I collagenase in D-Hanks balanced salt solution for 1 h at 37°C at 200 rpm. Then, the collagenase activity was neutralized by adding an equal volume of DMEM/F12 supplemented with 5% FBS, and the sample was pipetted up and down several times. The floating adipocytes were separated by centrifugation at 400 g for 5 min. The pellet was collected and filtered through a 70 μm cell strainer. The cells were plated in a 10 cm dish with growth medium (DMEM/F12 containing 5 ng/ml EGF, 5 ng/ml bFGF and GlutaMAX). Growth medium was replenished every three days until the cells reached 90% confluence, and the cells were then passaged with trypsin/EDTA solution. Adipogenic and osteogenic differentiation assays were performed to evaluate the potential for pluripotent differentiation. For adipogenic differentiation, cells were grown to 90% confluence and then changed to adipogenic differentiation medium 1 (DMEM supplemented with 5% FBS, 0.5 M IBMX, 1 μM dexamethasone, 1 μM rosiglitazone and 1.7 μM insulin) for three days. Next, the cells were incubated with adipogenic differentiation medium 2 (DMEM supplemented with 5% FBS and 1 μM rosiglitazone) for 8 days, and the medium was changed every three days. For osteogenic differentiation, cells were grown to 90% confluence and then changed to osteogenic differentiation medium (DMEM supplemented with 5% FBS, 0.01 μM 1,25-dihydroxyvitamin D3, 50 μM vitamin C and 10 mM β-sodium glycerophosphate). The osteogenic differentiation medium was changed every three days for a period of 27 days. ASCs from passage 4 to passage 10 were used in this study. Both types of cells were cultured in a 37°C incubator with 5% CO_2_.

### siRNA or plasmid transfection

Transfection was performed using jetPRIME transfection reagent according to the manufacturer’s protocol. OP9 cells were 50% or 80% confluent at the time of siRNA or plasmid transfection, respectively. The medium was replaced with adipogenic differentiation medium 24 h post-transfection. The siRNA sequence for mouse *Ndufa6* was 5ʹ-CGAGAAAUGUUCAUGAAGAAUTT-3ʹ, that of *Scd1* was 5ʹ-AGUUUCUAAGGCUACUGUCUUTT-3ʹ, and universal negative control siRNA (GenePharma, A06001) was used as a control. pcDNA3.1-*Scd1* was used for SCD1 expression in OP9 cells, and the pcDNA3.1 empty vector was used as a control.

### Oil red O staining

Following 8 days of adipogenic differentiation, cells were rinsed with PBS, fixed with 4% neutral buffered formalin at room temperature for 30 min, washed twice with PBS, stained with Oil Red O reagent for 15 min at room temperature, and then counterstained with haematoxylin solution. Cell images were captured using a Nikon Eclipse Ti2-U inverted microscope.

### Alizarin red staining

Alizarin red staining was performed based on the instructions of the Alizarin Red S staining kit. Cell images were captured using a Nikon Eclipse Ti2-U inverted microscope.

### Real time quantitative PCR (RT–qPCR)

An Ultrapure RNA Kit, HiScript® III 1st Strand cDNA Synthesis Kit and Hieff UNICON® qPCR SYBR Green Master Mix were used according to the manufacturer’s instructions. RT–qPCR was run at 95°C for 30 s and 60°C for 30 s for 40 cycles with a Roche LightCycler 480 II system. The primers used were: *Nudfa6*: 5ʹ-TCGGTGAAGCCCATTTTCAGT-3ʹ (forward), 5ʹ-CTCGGACTTTATCCCGTCCTT-3ʹ (reverse); *Scd1*: 5ʹ-TTCTTGCGATACACTCTGGTGC-3ʹ (forward), 5ʹ-CGGGATTGAATGTTCTTGTCGT-3ʹ (reverse); *Pparg*: 5ʹ-TCGCTGATGCACTGCCTATG-3ʹ (forward), 5ʹ-GAGAGGTCCACAGAGCTGATT-3ʹ (reverse); *Fabp4*: 5ʹ-AAGGTGAAGAGCATCATAACCCT-3ʹ (forward), 5ʹ-TCACGCCTTTCATAACACATTCC-3ʹ (reverse); *Fasn*: 5ʹ-AGAGATCCCGAGACGCTTCT-3ʹ (forward), 5ʹ-GCTTGGTCCTTTGAAGTCGAAGA-3ʹ (reverse); *C/ebpα*: 5ʹ-GCGGGAACGCAACAACATC-3ʹ (forward), 5ʹ-GTCACTGGTCAACTCCAGCAC-3ʹ (reverse); and *β-actin* 5ʹ-TGTTACCAACTGGGACGACA-3ʹ (forward), 5ʹ-CTGGGTCATCTTTTCACGGT-3ʹ (reverse). Gene expression changes relative to controls were determined using the 2^−ΔΔ^ method.

### Western blot

Cells were washed with phosphate-buffered saline (PBS) and lysed in RIPA buffer with 1X protease inhibitor cocktail. Protein samples were separated by SDS–PAGE and electrically transferred to PVDF membranes. TBST containing 5% skim was used to block the membranes for 1 h, and the membranes were washed 3 times with TBST. The membranes were incubated overnight at 4°C with primary antibodies. The next day, membranes were washed 3 times with TBST and incubated with HRP-conjugated secondary IgG antibody for 1 h at room temperature. Before imaging, the membranes were washed with TBST 3 times, and an ECL reagent kit was used to detect expressed proteins.

### Measurement of total triacylglycerol (TG)

A TG assay kit was used to measure the TG content, and total cellular protein was determined by a total protein assay kit according to the manufacturer’s protocol. The TG content was normalized to the total cellular protein.

### Fatty acid methyl ester (FAME) analysis

Cells were collected after adipogenic differentiation using RIPA buffer, lysed by ultrasound using a SONICS® (Sonics & Materials, Inc.) sonicator at a 20% amplitude setting (work 2 s and rest 3 s, 5 cycles). Total protein was measured using a total protein assay kit. Total lipid extraction was performed according to the method of Bligh and Dyer [44] using 14% boron trifluoride methanol (v/v) as the methylating agent [45]. Samples were quantified on a Q Exactive™ GC Orbitrap™ GC–MS/MS (Thermo Scientific) with a Rtx-Wax column (30.0 m × 0.25 mm, Restek, 12423). The temperatures of both the injection port and the detector were kept constant at 280°C. The column temperature was initially held at 40°C for 5 min, followed by an increase at a rate of 40°C/min to 120°C, then to 190°C at 10°C/min for 5 min, and then to 230°C at 5°C/min for 7 min; the total time was approximately 34 min for all fatty acid peaks. Peaks were identified by comparing retention times with known standards (Sigma Chemical). Individual fatty acids were quantified by reference to the internal standard (C17:0). Subsequently, the sample was normalized to total cellular protein.

### Lipidomic analysis

Cell samples were prepared as described above. Total lipid extraction was performed according to the method of Matyash et al. [46]. Lipidomic analysis was performed on a Q Exactive Plus mass spectrometer (Thermo Scientific) equipped with a Vanquis UHPLC (Thermo Scientific). Mass spectrometry (MS) and tandem mass spectrometry (MS/MS) data were acquired in positive and negative ion modes. Before sample infusion into the MS, 1 µl of lipid mixture was separated at 40°C on an ACQUITY UPLC BEH C18 column (1.7 μm, 2.1 × 100 mm, Waters). For both the positive and negative modes, mobile phase A was 10 mM ammonium acetate in acetonitrile:H_2_O (60:40), and mobile phase B was 10 mM ammonium acetate in isopropyl alcohol:acetonitrile (90:10). Liquid chromatography gradients were as follows: 0–3 min, 40% B-40% B; 3–20 min, 40% B-95% B; 20–22.5 min, 95% B-95% B; and 22.5–23 min, 95%-40% B. The flow rate was 0.25 ml/min. The quality control (QC) samples were prepared by mixing different samples, and the order of QC samples was evenly distributed among samples. Lipids were identified using LipidMap (www.lipidmaps.org), and statistical analysis of lipid profiling of samples was performed using SIMCA (14.1) software.

### cDNA library construction and RNA sequencing (RNA-seq)

For cDNA library construction, first-strand cDNA was reverse-transcribed from mRNA and further converted into double-strand cDNA. Then, the double-stranded cDNA was resuspended in Tn5 transposase reaction mix, followed by digestion and tagmentation. Adapters and primers were synthesized according to published Illumina sequences. Enrichment PCR was performed using HIFI PCR Mix for NGS. The PCR amplification procedure was: 72 ℃ for 5 min, 98 ℃ for 30 s, and 25 cycles of 98 ℃ for 10 s, 65 ℃ for 30 s, 72 ℃ for 1 min, and 72 ℃ for 10 min. Libraries were quantified using Agilent 2100 BioAnalyzer, and sequenced using Illumina NovaSeq instrument (Sequencing was performed by GENEWIZ Biotech). Sequencing data were analysed using STAR (http://code.google.com/p/rna-star/) and R studio (R studio Inc.). The differentially expressed genes were defined as genes with a P value < 0.05 and a fold change ≥ 1.5. Gene ontology (GO) analysis was performed using Metascape (http://metascape.org). Heatmap was generated using Tbtools software (https://github.com/CJ-Chen/TBtools).

## Supplementary Material

Supplemental Material

## Data Availability

The datasets used and/or analysed during the current study are available from the corresponding author on reasonable request.

## Declarations

This study received ethical approval from the ethical committee of Jiangnan University (JN.No20210530c0601230[153]).

## List of abbreviations

NADH:ubiquinone oxidoreductase subunit A6: *Ndufa6*

stearoyl-CoA desaturase 1: *Scd1*

adipose-derived stem cells: ASCs

fatty acid synthase: FASN

acetyl-CoA carboxylases: ACC
