# Supplementary figures and images for: Ndufa6 regulates adipogenic differentiation via Scd1
Source: Adipocyte. 2021 Dec 7;10(1):646–57. doi: 10.1080/21623945.2021.2007590 (PMC8654480; doi:10.1080/21623945.2021.2007590)

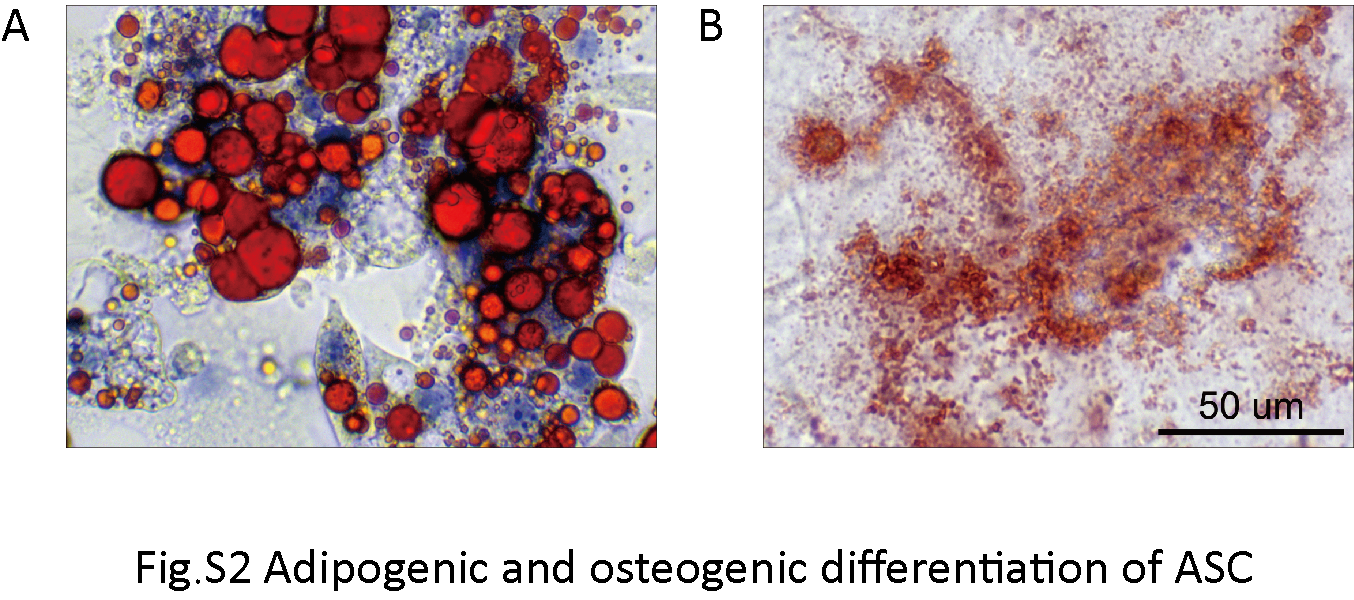

Supplement: Supplemental Material [file KADI_A_2007590_SM5538.zip › supplementary/Fig. 2S.tif]

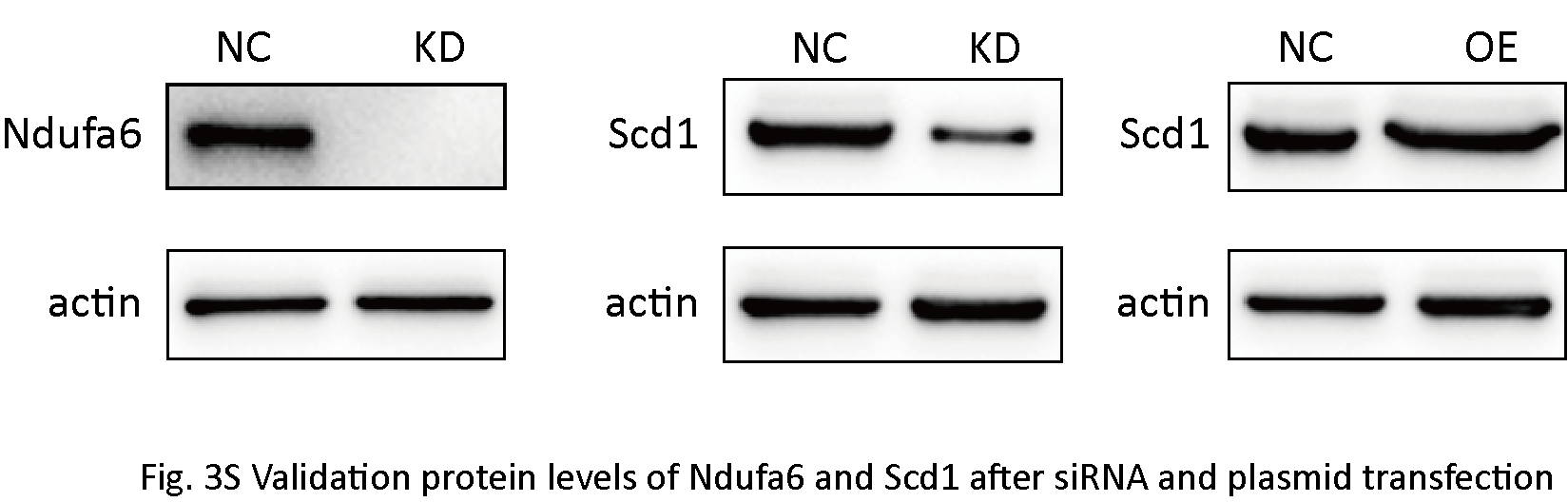

Supplement: Supplemental Material [file KADI_A_2007590_SM5538.zip › supplementary/Fig. 3S.tif]

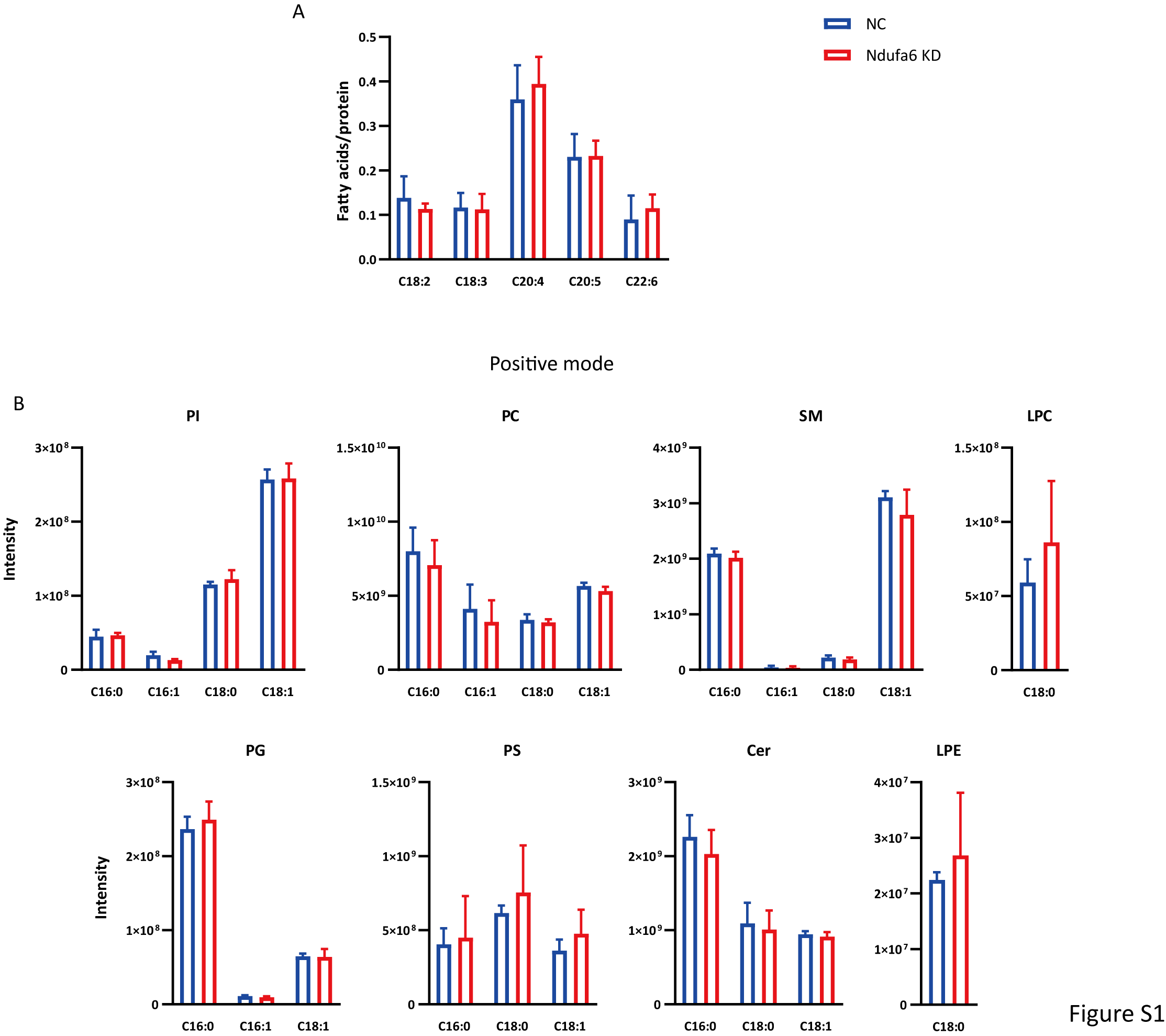

Supplement: Supplemental Material [file KADI_A_2007590_SM5538.zip › supplementary/Fig.S1.tif]
